# Method development and validation for microscopic measurement of the fluorescence spectrum of sedimentary organic matter in geologic samples

**DOI:** 10.1016/j.mex.2026.104003

**Published:** 2026-06-13

**Authors:** Kavin Wee Keat Siaw, Paul C. Hackley

**Affiliations:** a U.S. Geological Survey, Reston, VA, United States; b Brigham Young University – Idaho, Rexburg, ID, United States

**Keywords:** Organic petrology, Fluorescence spectroscopy, Method validation, Thermal indices, Fossil fuels, Burial history, Oil window

## Abstract

The goal of this study was to develop and validate a standard operating procedure for measuring the fluorescence emission of sedimentary organic matter (SOM), a proxy used to assess thermal maturity conditions in petroliferous basins. We tested the performance of a spectrometer integrated with an epi-fluorescence imaging microscope, and evaluated instrument calibration, dark-current correction, time duration for instrument stabilization, light delivery optimization, and the number of measurements necessary to optimize precision. These tests were used to guide instrument operation, ensure measurement accuracy, and characterize performance limitations of the system as SOM transitions to higher thermal maturity with accompanying signal deterioration. Adhering to these newly established guidelines enhances the reproducibility of fluorescence spectroscopy measurements. More importantly, this work endeavors to reach the overall objective of demonstrating the utility of fluorescence spectroscopy for evaluating the thermal maturity of SOM.

Method for fluorescence spectroscopy of sedimentary organic matter.

Internal calibration via diffuse halogen light reflected from polytetrafluoroethylene (PTFE).

Evaluates emission response via *λ*_max_ and red-green and blue-green quotients.

## Graphical abstract

Fluorescence spectra from sporinite (sporinite example shown in epi-fluorescence) in subbituminous coal from North Dakota, USA

**Figure:**
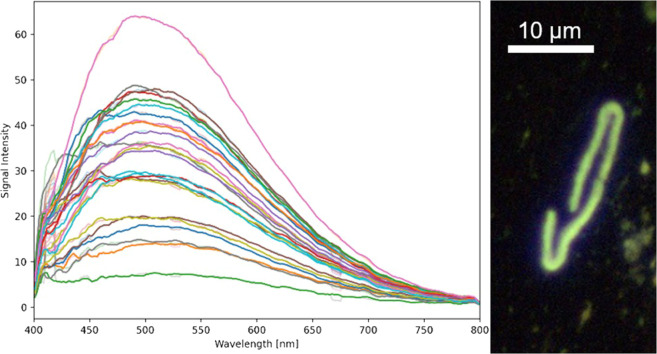

Specifications table

**Table:** 

Subject area	Energy
More specific subject area	Organic Petrology
Name of your method	Spectral Fluorescence Measurement of Sedimentary Organic Matter
Name and reference of original method	Baranger, R., Martinez, L., Pittion, J.-l., Pouleau, J., 1991. A new calibration procedure for fluorescence measurements of sedimentary organic matter. *Organic Geochemistry* 17, 467–475, among other method publications
Resource availability	https://hilgers.com/

## Background

The U.S. Geological Survey (USGS) conducts periodic assessments of the oil and natural gas resources of the United States as well as global hydrocarbon-based energy resource estimates [[1], [2], [3]]. For unconventional source-rock reservoirs, thermal proxies—such as the fluorescence emission of sedimentary organic matter (SOM)—can be used to constrain the boundaries of oil and gas accumulations [4]. The fluorescence emission wavelength of SOM shifts to longer wavelengths, i.e., red shifts, with increasing aromatization throughout burial in the shallow crust of the Earth [5]. Therefore, measurement of fluorescence parameters such as *λ*_max_ (the wavelength of maximum intensity emission), and red-green (R/G) and blue-green (B/G) quotients (i.e., integrated signal from red: 630–670 *nm*; green: 545–555 *nm*; and blue: 460–470 *nm)* allows the use of fluorescence color as a thermal proxy in the exploration for and assessment of hydrocarbon-based energy resource accumulations in petroliferous sedimentary basins [6]. Although SOM fluorescence emission has been measured for half a century [7], reproducible measurement in interlaboratory studies has been difficult to achieve due to the lack of a standardized test method and access to appropriate reference materials [8,9]. Here, we report the development of a microscope-spectrometer method to measure fluorescence emission of SOM and method validation for a standard operating procedure used in the USGS Organic Petrology Laboratory (OPL) in Reston, Virginia. The method was developed in the context of an internal quality management system for laboratories at USGS with the goal of providing data of known and documented quality [10]. This work reaches toward the overall broader goal of standardization of fluorescence measurement of SOM, an effort convened under the auspices of the International Committee for Coal and Organic Petrology (https://www.iccop.org/workinggroup/standardization-of-fluorescence-measurement-wg/).

## Method details

**Fig. 1:**
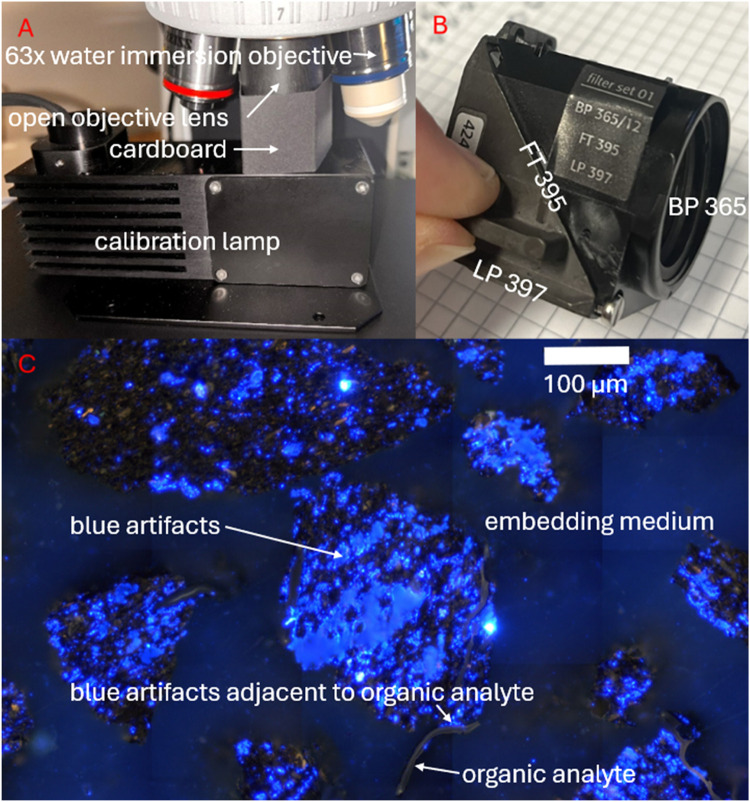
(A) Calibration lamp positioned on the microscope stage with open objective lens above. A black cardboard light barrier is inserted between the lens and the calibration lamp to eliminate stray light. (B) Filter Set 01 includes bandpass excitation filter (BP 365/12), dichroic mirror (FT 395), and long-pass emission filter (LP 397). (C) Mosaic scan of polished fragments of shale sample embedded in PMMA showing intense blue artifact emission.

This method uses a Zeiss Axio lmager M2m microscope (https://www.zeiss.com/microscopy/us/products/light-microscopes/widefield-microscopes/axio-imager-2-for-materials.html) coupled to a desktop TEC5 spectrometer (https://tec5.com/en/). The system is controlled by a personal computer Hewlett Packard Workstation Z2 G9 operating the Hilgers DISKUS 1600 software (refer to https://hilgers.com/DISKUS-SOFTWARE/ for system information) for image acquisition, microscope control, measurement and management. A Hitachi HVF-203GV digital camera is controlled through the Hilgers DISKUS software for location, imaging, and visualization of microscopic analyte materials. The microscope-spectrometer system employs a customized Hilgers calibration lamp which uses the diffuse light of a halogen bulb reflected from polytetrafluorethylene (PTFE) to create a consistent reproducible spectrum used as an internal calibration. PTFE is an inert fluoropolymer, i.e., hydrophobic and with a high temperature rating, which is widely used in applications requiring a non-reactive surface, e.g., insulations, lubrications, coatings, and linings [11]. The spectrum of the PTFE calibration lamp is measured at each use of the microscope-spectrometer and is compared to its prior measurement which is stored in the DISKUS software to mitigate against instrument drift and to ensure measurement stability. All measurements were conducted under controlled optical conditions, using a 63*x* magnification water immersion objective with 0.9 numerical aperture and ultraviolet excitation light delivered from a Hg gas-discharge excitation lamp through a bandpass (BP) 365/12 filter set, incorporating a short-pass dichroic filter (FT 395), and a long-pass emission filter (LP 397) (Fig. 1B). Data are contained in tables in this report and available in Siaw et al. [12].

### Instrument preparation time

To optimize preparation time, this study evaluated various heating durations for the calibration lamp prior to performing spectral measurement (measured immediately, after 5 min, 10 min, 15 min, and 30 min warm-up). Each measurement was conducted under consistent environmental conditions, with a black cardboard light barrier between the objective lens and the calibration lamp (Fig. 1A). This test allows for evaluating whether signal intensity or structure is significantly influenced by instrument preparation time and determines if a standardized waiting period is necessary to ensure consistent calibration performance.

### Calibration conditions and light cable diameter

This study evaluated the influence of different calibration environments through measurement in complete darkness and under ambient laboratory light. Measurements were also made in the presence and absence of a black cardboard light barrier placed between the objective lens and the calibration lamp to test the effectiveness of the barrier in reducing stray light interference. Calibration conditions were also tested in combination with different fiber-optic cable diameters (100 *μm* and 200 *μm*) to determine if calibration consistency was affected by collection geometry. Smaller-diameter cables (e.g., 100 *μm*) offer the potential for higher spatial resolution, which may be advantageous when targeting fine-grained structures or heterogeneous matrices. However, they capture a lower photon count which reduces signal-to-noise in contrast to larger-diameter cables (e.g., 200 *μm*) which enable greater light throughput, improving signal strength, but which also may sacrifice spatial specificity and introduce averaging effects from surrounding non-analyte media.

### Dark current correction frequency

This study investigated the influence of dark current correction frequency on the accuracy and consistency of fluorescence spectral data. Dark current correction is essential for isolating the inherent electrical signal (i.e., noise) generated by the CCD (charge-coupled device) detector in the spectrometer in the absence of incident light, i.e., without any incoming photon counts [13]. This correction is critical for eliminating systematic bias, particularly in low-signal measurements, and improving the signal-to-noise ratio. Dark current correction was performed immediately after powering on the spectrometer, and again after a ten-minute warm-up period. In both scenarios, calibration reference spectra were acquired to evaluate the spectral peak position (*λ*_max_) and the corresponding red-green-blue (RGB) intensity quotients. These data were then analyzed to assess whether the timing of dark current correction significantly affected calibration or introduced variability into the derived spectral metrics.

### Stray light analysis

The quality of fluorescence spectra can be significantly influenced by stray light sources, including interference from the embedding medium, ambient room light, or non-analyte sample features, e.g., fluorescent minerals adjacent to analyte organic matter [14]. These sources of interference can distort the spectral signal, introduce false peaks, or shift spectral intensities, thereby compromising the accuracy of measurement. This study systematically investigated the influence of various stray light sources on peak position and RGB fluorescence intensity quotients.

#### Embedding medium

The sample embedding medium poly(methyl methacrylate) (abbreviated PMMA) used in the USGS OPL can emit strong fluorescence in the blue region (400–480 nm) when exposed to ultraviolet excitation. This emission can obscure or overlap with signals from the organic analyte material in the sample. This study specifically targeted sample regions near the embedding medium, comparing fluorescence spectra collected adjacent to the medium with those taken from more isolated regions.

#### Ambient light

Ambient room lighting, particularly from overhead fluorescent or LED sources, can introduce background illumination that affects the calibration spectra collected by the spectrometer. To investigate this effect, calibration measurements were performed in the windowless OPL, with and without the room ceiling lights on (the main source of ambient light in OPL).

#### Sample artifacts

We observed intense blue emission artifacts from sample preparation (Fig. 1C) in select samples, which were hypothesized to result from pocket-like voids or holes within the sample that were filled with PMMA. When exposed to ultraviolet excitation, these pockets of PMMA may strongly reflect or scatter the excitation signal, producing bright, localized blue emission artifacts that interfere with the detection of analyte signal from the target organic matter. To investigate their impact, fluorescence measurements of sample analyte were conducted at multiple positions near and away from blue artifacts.

### Number of analyte measurements

To optimize measurement precision, this study investigated the appropriate number of analyte measurements required for reliable analysis. A systematic approach was designed to evaluate the stability of repeated measurements across shale and coal samples by calculating the coefficient of variation (CV) for each dataset. The coefficient of variation [15] is a normalized measure of dispersion expressed as a percentage:

(1) $$CV=\left(\frac{\sigma }{\mu }\right)\times \;100$$

where *σ* is the standard deviation and *μ* is the mean of the measurements. This metric enables direct comparison of variability across datasets with different scales. In this study, CV was reported in decimal format (i.e., without multiplying by 100) as shown in eq. (2). A lower CV value indicates higher consistency and conversely a higher CV suggests greater variability.

(2) $$CV=\frac{\sigma }{\mu }$$

### Statistical analysis

To evaluate whether the number of measurements affected the stability of the spectral measurements, the coefficient of variation (CV) was analyzed for both *λ*_max_ and the R/G quotient. The CV was used as a measure of relative variability, where a lower CV indicates more stable and repeatable measurements. Statistical analyses were performed separately for coal and shale samples because the experimental designs were different.

#### One-Way ANOVA

A one-way analysis of variance (ANOVA) was first performed to determine whether the mean CV differed among the different numbers of measurements. For the coal samples, the measurement groups were 5, 10, 15, 20, and 25 measurements. For the shale samples, the measurements groups were 5, 10, and 15 measurements.

The null hypothesis for the ANOVA was that the mean CV was equal across all measurement-number groups, and the alternative hypothesis was that at least one group mean was different at the 5% significant level:

$H_{0}=\mu_{5}=\mu_{10}=\mu_{15}=\mu_{20}=\mu_{25}$ for the coal samples.

$H_{0}=\mu_{5}=\mu_{10}=\mu_{15}$ for the shale samples.

*H_a_*: At least one measurement group has a different mean CV, and *α*=0.05.

If the ANOVA p-value was <0.05, the number of measurements was considered to have a statistically significant effect on the CV. If the p-value was greater than or equal to 0.05, there was no statistically significant evidence that the CV differed among the measurement groups. The ANOVA was used as an overall test to determine whether measurement number had a general effect on variability. However, because the main goal was to evaluate whether 10 measurements were sufficient, additional pairwise comparisons were performed between the 10-measurement group and the other measurement groups.

#### Pairwise comparisons

To directly evaluate whether 10 measurements were statistically comparable to the other measurement numbers, pairwise *t*-tests were performed using the 10-measurement group as the reference. The comparisons were: 10 *vs* 5, 10 *vs* 15, 10 *vs* 20, 10 *vs* 25 for the coal samples and 10 *vs* 5, 10 *vs* 15 for the shale samples. For each comparison, the general null and alternative hypotheses were:

$$H_{0}:\mu_{10}=\mu_{other},\;H_{a}:\mu_{10}\neq \mu_{other}\;\&\;\alpha =0.05$$

Where *μ*_10_ is the mean CV from the 10-measurement group and *μ*_other_ is the mean CV from another measurement group.

A non-significant result indicates that the 10-measurement group was not statistically different from the comparison group. In particular, for the coal samples, non-significant differences between 10 measurements and the larger measurement groups, 15, 20 and 25 measurements, supports the conclusion that increasing the number of measurements beyond 10 did not significantly improve measurement stability.

#### Paired t-Tests for coal samples

For the coal samples, paired *t*-tests were used because the same coal samples were measured repeatedly using different numbers of measurements. Therefore, the CV values at 5, 10, 15, 20, and 25 measurements were related rather than independent. For each paired comparison, the difference was calculated as:

(3) $$d_{i}=CV_{10,i}-CV_{other,i}$$

where *i* represents the same coal sample. The paired *t*-test evaluates whether the mean paired difference is significantly different from zero:

$$H_{0}:\mu_{d}=0,\;H_{a}:\mu_{d}\neq 0\;\&\;\alpha =0.05$$

where *μ_d_* is the mean difference between the 10-measurement CV and the CV from another measurement number. This test was appropriate for the coal samples because each sample served as its own reference across the different measurement-number groups.

#### Independent welch t-Test for shale samples

For the shale samples, the independent Welch *t*-test was used for the pairwise comparisons. Although the same shale sample was used, the measurements were collected under different conditions and at different locations. That is, these measurements were not matched sample-by-sample in the same way as the coal measurement groups. Welch’s independent *t*-test was selected instead of the standard independent *t*-test because it does not assume equal variances between the two groups. This makes it more appropriate for small datasets and for comparisons where variability may differ between measurement conditions. For each shale comparison, the null and alternative hypotheses were:

$$H_{0}:\mu_{10}=\mu_{other},\;\;H_{a}:\mu_{10}\neq \mu_{other}\;\&\;\alpha =0.05$$

Where *μ*_15_ is the mean CV for the 10-measurement group and *μ_other_* is the mean CV for the comparison group.

#### Holm correction for multiple comparisons

Because multiple pairwise comparisons were performed within each dataset, the Holm correction was applied to control the family-wise error rate. This correction was necessary because performing several statistical tests increases the probability of obtaining a false significant result by chance. For each dataset, the raw p-values from the pairwise comparisons were ranked from smallest to largest and adjusted using the Holm method. For *m* comparisons, the ranked p-values were adjusted according to:

(4) $$p_{adj,\left(i\right)}=p_{\left(i\right)}\left(m-i+1\right)$$

Where *p*_(_*_i_*_)_ is the *i* th smallest p-value, *m* is the total number of comparisons, and *i* is the rank of the p-value after sorting. Adjusted p-values were capped at 1.000 and interpreted using *p_Holm_*<0.05 as the criterion for statistical significance, and the Holm correction was applied separately within each dataset.

#### Criteria for determining whether 10 measurements were sufficient

Ten measurements were considered sufficient if the CV values obtained from 10 measurements were not significantly different from those obtained using other measurement quantities. That is, if 10 measurements did not differ significantly from 15, 20, or 25 measurements, then increasing the number of measurements beyond 10 was interpreted as not producing a statistically significant improvement in measurement stability. For the shale samples, 10 measurements were compared with 5 and 15 measurements using the independent Welch *t*-test. These comparisons were used to evaluate whether the 10-measurement results were statistically different from the other measurement-number groups under the shale measurement conditions. This approach allowed 10 measurements to be evaluated as a statistically comparable and practical measurement number for the analysis.

### Interpolation

**Fig. 2:**
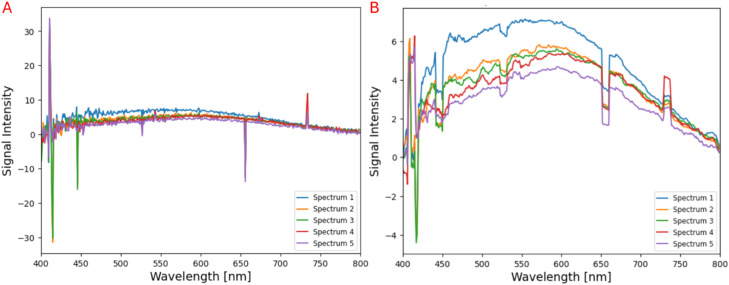
A. Spectral artifacts—characterized by extreme peaks and troughs—were occasionally observed in the raw spectra, potentially affecting the calculation of ***λ*_max_** and RGB quotients. B. The same spectra after applying a moving average in the software with a window size of 9 nm, which further highlighted the persistence of these anomalies, supporting the occasional need for targeted interpolation.

The spectrometer occasionally produced signal artifacts of unknown cause characterized by extreme peaks and troughs (Fig. 2A-B), which potentially interfere with accurate determination of *λ*_max_ and RGB quotients. To address these signal artifacts, interpolation was applied to the raw spectral data prior to calculation of *λ*_max_ and RGB quotients. Any point identified as an artifact was replaced by the average of the adjacent preceding and following data points.

The resulting *λ*_max_ values and RGB quotients—along with their respective standard deviations—were compared before and after interpolation. These values were also assessed against those calculated directly by moving average in the Fossil spectrometer software to determine whether interpolation enhanced accuracy or introduced systematic bias. Interpolation was applied only in cases where spectrometer artifacts directly impacted the parts of the spectra used to compute *λ*_max_ values and RGB quotients.

### BAM standard secondary validation

In addition to the primary calibration procedure using the PTFE reflection lamp, a secondary validation was performed using a certified reference material, BAM-F012, which is a glass-based multi-emitter fluorescence standard comprised of a lanthanum phosphate glass doped with luminescent rare earth element metal ions [Ce(+III), Tb(+III), and Eu(+III)] [16]. This test compared the measured emission spectrum with the known reference emission peaks of BAM-F012. Ten repeated measurements were acquired on the reference material, and the averaged results were evaluated against the spectral profile of the certified reference material to assess consistency and accuracy.

## Method validation

### Instrument preparation time

**Fig. 3:**
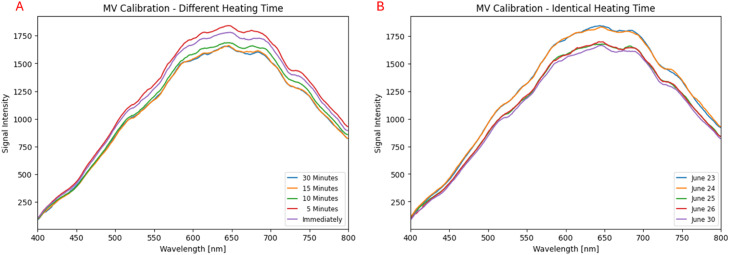
A. Calibration spectra collected with different heating durations for the calibration lamp (0, 5, 15, and 30 min). B. Calibration spectra with identical heating durations (5-minute heating period on different days). MV, moving average applied (**9*nm***).

Heating the calibration lamp for five different durations demonstrated a visually consistent spectral structure across all conditions (Fig. 3A). This indicated that the duration of lamp heating prior to calibration does not affect a stable baseline reference. However, an approximate 10% range in signal intensity was observed dependent on the time interval between powering on the calibration lamp and acquiring the calibration spectra. To further evaluate this effect, calibration spectra collected after different heating durations (Fig. 3A) were compared with those obtained after a consistent heating time of five minutes (Fig. 3B).

It is evident that similar magnitude (∼10%) daily variations in calibration spectra signal intensity occur even when the calibration lamp heating time is kept consistent. Thus, the heating time duration alone does not have a direct effect on the calibration and data quality for fluorescence color measurements. Nevertheless, following Hilgers Technisches Buero [17], a five-minute heating period for the calibration lamp is recommended as standard daily operating protocol.

### Calibration conditions and light cable diameter

**Fig. 4:**
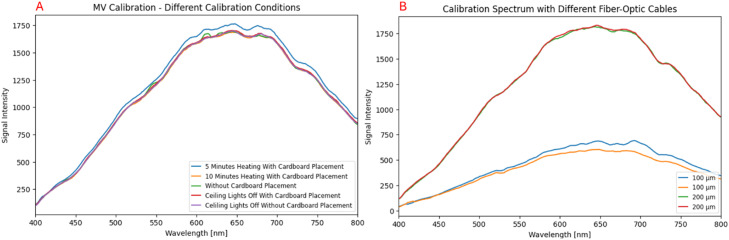
A. Calibration spectra collected under different environmental conditions, showing a significant stray light effect from ambient room light at ∼**600*nm*** when the black cardboard light barrier is not placed between the objective and the calibration lamp. B. Calibration spectra collected using two different fiber-optic cable diameters (**100*μm*** and **200*μm***) showing a substantial difference in signal intensity. MV, moving average applied (**9*nm***).

When the ceiling lights are on and the light barrier is not placed between the objective and the calibration lamp, an unexpected peak appeared near 600 *nm* in the calibration lamp spectrum (Fig. 4A). This indicates that stray room light can introduce spectral artifacts if not properly managed. The test provides strong evidence that placing a light barrier between the objective and the calibration lamp helps to reduce such interference and improves calibration consistency. When the ceiling lights are off, the presence or absence of the light barrier showed no effect on the overall spectral profile. Hence, performing calibration and sample measurement with the ceiling lights off is recommended in all cases to prevent ambient room light from influencing measurement results. The type of fiber-optic cable used during calibration also affects both the outline and intensity of the spectrum. When switching between fiber-optic cables (100 *μm* vs. 200 *μm*), a notable change in signal intensity is observed with the intensity approximately 3–3.5*x* greater at *λ*_max_ with the 200 *μ*m fiber (Fig. 4B), similar to the expected 4-fold increase in intensity if intensity scales linearly with the cross-sectional area of the fiber optic. This observation highlights the importance of recalibration when fiber-optic cables are changed.

**Fig. 5:**
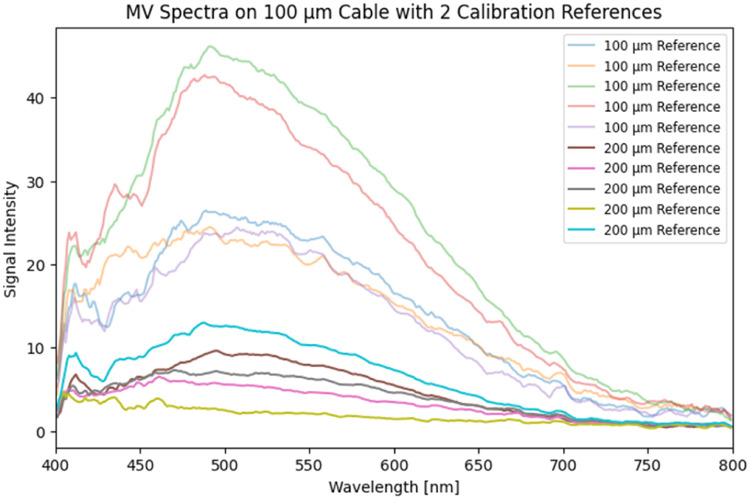
Moving average spectra (window size = **9*nm***) collected on a coal sample using **100*μm*** fiber-optic cable with two calibration references (**100*μm*** and **200*μm***). Faded lines represent calibration using the **100*μm*** reference; solid lines show calibration using the **200*μm*** reference. A clear intensity difference is observed.

The effect of fiber-optic cable diameter on spectral calibration and signal quality was evaluated using 100 *μm* and 200 *μm* fiber-optic cables. Calibration spectra were compared by applying two different reference conditions—one matched to the cable used and one mismatched. As shown in Fig. 5, spectra collected with the 100 *μm* fiber-optic cable and normalized using the correct 100 *μm* reference yield significantly different results than if the data were normalized using the incorrect 200 *μm* reference. This discrepancy is attributed to spectral subtraction during calibration; if the reference spectrum does not match the characteristics of the light cable used during data acquisition, the subtraction impacts the resulting signal intensity, e.g., greater signal intensity is subtracted from the 200 *μm* reference.

**Fig. 6:**
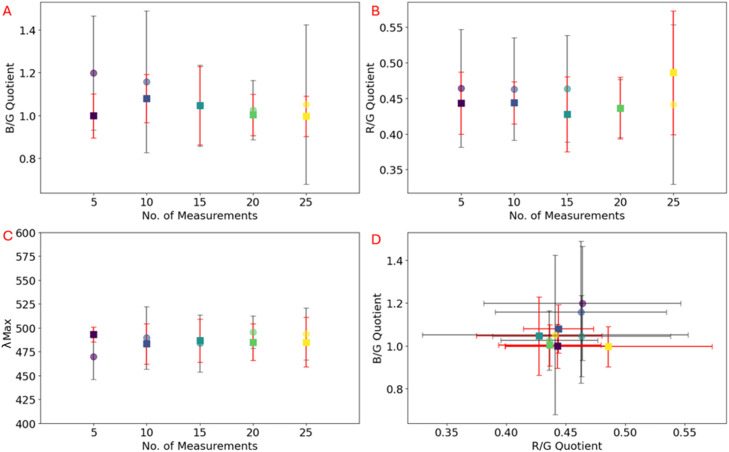
RGB quotients and ***λ*_max_** for multiple measurements of a coal sample using **100*μm*** fiber-optic cable with two calibration references. Faded grey error bars with circular symbols show standard deviation using the mismatched **200*μm*** cable calibration reference; solid red error bars with square symbols correspond to the matched **100*μm*** cable reference. Different color symbols correspond to 5, 10, 15, 20, and 25 measurements. The **100*μm***-matched reference provides more accurate values as indicated by lower standard deviation in all panels. A. B/G quotient. B. R/G quotient. C. ***λ*_max_**. D. B/G quotient versus R/G quotient.

This mismatch may result in spectral artifacts or reduce intensity even when *λ*_max_ appears within the expected range. These discrepancies are further demonstrated in Fig. 6, which shows the RGB quotients and *λ*_max_ from a set of coal measurements taken with the 100 *μm* cable under both reference conditions, i.e., generated with 100 *μm* and 200 *μm* fiber-optic cables. These data affirm that it is necessary to match experiment conditions to the reference conditions.

**Fig. 7:**
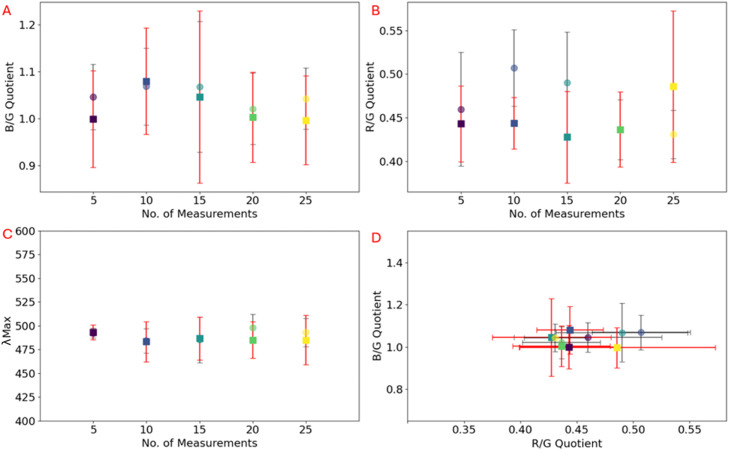
RGB quotients and ***λ*_max_** from measurements using both **100*μm*** and **200*μm*** fiber-optic cables, each paired with their corresponding calibration references. Faded grey error bars with circular symbols show standard deviation for data from **200*μm*** cable and reference; solid red error bars with square symbols plot standard deviation for data from **100*μm*** cable and reference. Different color symbols correspond to 5, 10, 15, 20, and 25 measurements. Results indicate consistent calibration when references match the measurement cable, showing tighter placement and lower standard deviation relative to mismatched data shown in Fig. 6. A. B/G quotient. B. R/G quotient. C. ***λ*_max_**. D. B/G quotient versus R/G quotient.

When comparing the results using both 100 *μm* and 200 *μm* fiber-optic cables with their corresponding references, the measurements appear consistent, as shown in Fig. 7. Therefore, when performing measurements, it is critical that the calibration be conducted using the corresponding reference. In general, and especially in samples with high mineral content, higher thermal maturity, and lower fluorescence intensity, the 200 *μm* cable may be preferable due to its higher light throughput and stronger (approximately 3–4x) signal return.

### Dark current correction frequency

To assess the impact of dark current correction frequency on calibration spectra, measurements of the calibration lamp were collected under various environmental conditions using different timing protocols for dark current correction. As summarized in Table 1, only the ‘Immediately’ and ‘After 10 Minutes’ conditions used their corresponding dark current references. All other conditions used the ‘After 10 Min’ dark current correction to isolate environmental effects from dark current variability.

**Table 1:** RGB quotients and ***λ*_max_** values of the calibration lamp under different dark current correction and ambient light environmental conditions. Only the ‘Immediately With Light Barrier’ (measured immediately after power on using the black cardboard light barrier) and ‘After 10 Min With Light Barrier’ rows used matching dark current measurements; all other conditions used the ‘After 10 Min With Light Barrier’ dark current reference to isolate the effect of environmental factors.

Conditions	B/G Quotient	B/G std	R/G Quotient	R/G std	λ_max_	λ_max_ std
Immediately With Light Barrier	2.29	0.01	3.19	0.02	647	1.58
After 10 Min With Light Barrier	2.29	0.02	3.17	0.03	646	2.07
Without Light Barrier	2.30	0.02	3.13	0.02	644	2.35
Dark Room With Light Barrier	2.28	0.03	3.15	0.05	644	2.30
Dark Room Without Light Barrier	2.27	0.02	3.15	0.04	644	1.44
15 Min With Light Barrier	2.27	0.02	3.16	0.04	646	0.55

The results demonstrate that dark current correction does not significantly influence the calculated RGB quotients or the *λ*_max_ values of the calibration lamp. While some variation in lamp parameters is present, these remain within standard deviation, confirming that electronic noise is stable on the spectrometer and the frequency of dark current collection is not critical for maintaining calibration stability. Hence, it is proposed to perform dark current correction once a month (every 30 days) as suggested by the instrument vendor [17].

### Stray light analysis

#### Embedding medium

**Fig. 8:**
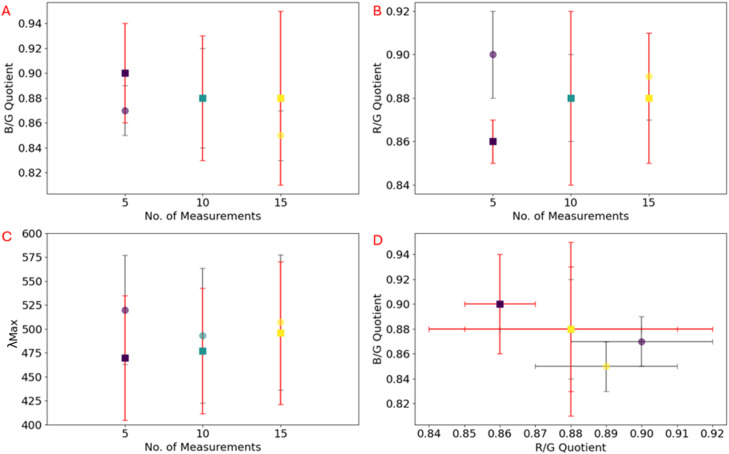
Fluorescence parameters from testing the influence of embedding media. The solid red error bars with square symbols show standard deviation of measurements taken at the edge of the sample near the embedding medium, and the faded grey error bars with circular symbols show standard deviation of measurements taken at the center of sample fragments away from the embedding medium. Different color symbols correspond to 5, 10, and 15 measurements. A. B/G quotient. B. R/G quotient. C. ***λ*_max_**. D. B/G quotient versus R/G quotient.

**Fig. 9:**
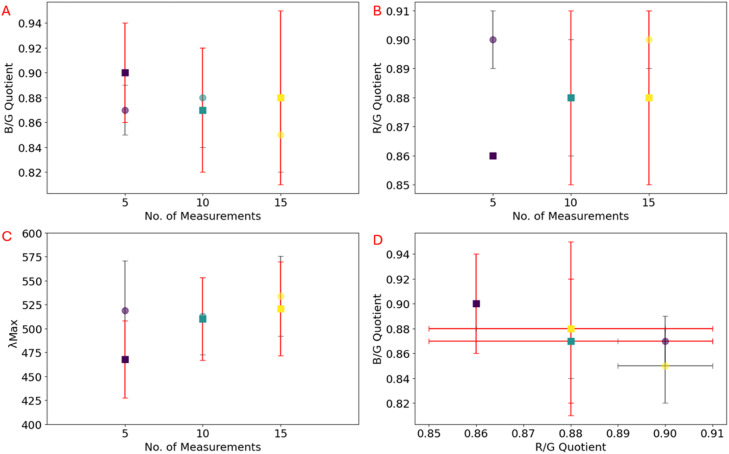
Fluorescence parameters from testing the influence of embedding media after interpolation using spectra >**450*nm***. The solid red error bars with square symbols show standard deviation of measurements taken at the edge of the sample near the embedding medium, and the faded grey error bars with circular symbols show standard deviation of measurements taken at the center of sample fragments away from the embedding medium. Different color symbols correspond to 5, 10, and 15 measurements. A. B/G quotient. B. R/G quotient. C. ***λ*_max_**. D. B/G quotient versus R/G quotient.

Fluorescence spectra collected near the embedding medium—specifically at the edge of the sample fragments adjacent to the binder—showed an increase in signal intensity between 400–450 *nm*. While the overall spectral shape remained consistent at wavelengths >450 *nm*, the elevated signal observed near the embedding medium may result in an inaccurate and reduced *λ*_max_ determination. Figure highlights that measurements taken near the embedding medium produced fluorescence parameter values accompanied by higher standard deviations. Moreover, average B/G quotients were higher in 2 of 3 measurement groups (5 and 15 measurements, Fig. 8A) and average *λ*_max_ values were lower in 3 of 3 measurement groups (Fig. 8C). To avoid any influence of the embedding medium, the evaluation window for *λ*_max_ detection was restricted to >450 *nm*, resulting in more consistent outcomes, i.e., higher measurement precision as demonstrated in Fig. 9A-D and consistent average *λ*_max_ and B/G quotient when evaluating measurements taken near and distant from the embedding medium. Overall, it is advisable to collect data from the part of the sample that is away from the embedding medium, e.g., embedding medium not present in the closed field diaphragm. Nevertheless, as long as *λ*_max_ occurs within the 450–800 *nm* bandwidth, *λ*_max_ is considered reliable.

#### Room ceiling light

**Fig. 10:**
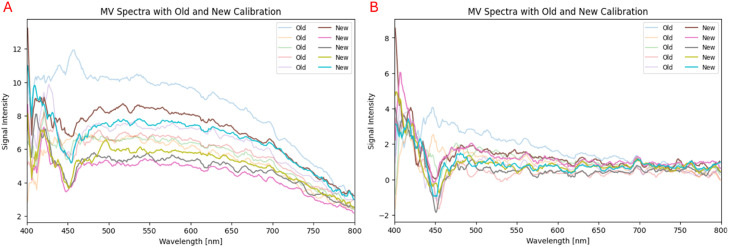
A-B. Spectra taken in two different calibration conditions on two shale samples. The faint spectra (‘Old’) show data from organic analyte material using a calibration completed with ceiling lights on and the solid line spectra (‘New’) show data collected using a calibration completed with ceiling lights off. In every case, the ‘Old’ and ‘New’ spectral pairs were collected at the same location in the same organic matter analyte fragment using a location memorization feature of the software.

Based on the observation of ambient room light impacting calibration spectra (Fig. 4A), data collection was performed from the same organic analyte locations using calibrations conducted with the ceiling lights on and off. Although this test may have been impacted by electronic noise, the structure of the collected spectra appeared to illustrate notable differences in certain replicate measurements, as illustrated in Fig. 10 from two shale samples.

**Fig. 11:**
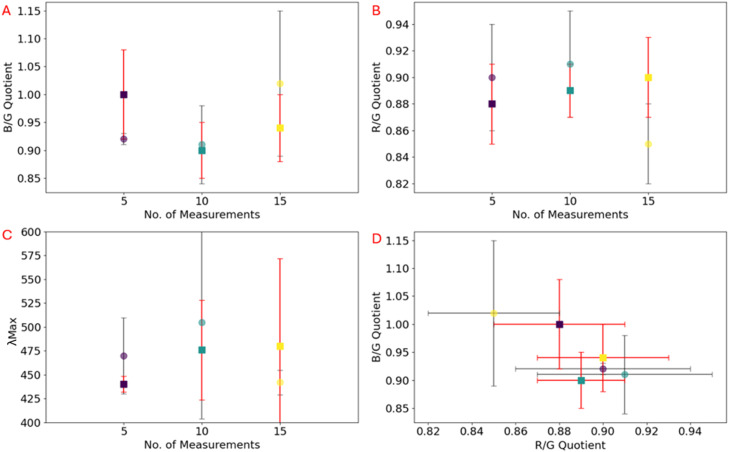
Fluorescence parameters from testing the influence of ambient light. The solid red error bars with square symbols show standard deviation of measurements taken with the room lighting off (calibration with room lighting off), and the faded grey error bars with circular symbols show standard deviation of measurements taken with the room lighting on (calibration with room lighting on). Different color symbols correspond to 5, 10, and 15 measurements. A. B/G quotient. B. R/G quotient. C. ***λ*_max_**. D. B/G quotient versus R/G quotient.

In addition, measurement precision generally improved for *λ*_max_ and RGB quotients when measurements were conducted in a dark environment using a reference calibration also performed in darkness (Fig. 11 Hence, these tests confirm that calibration and data collection should be performed in a dark room.

#### Blue artifacts

**Fig. 12:**
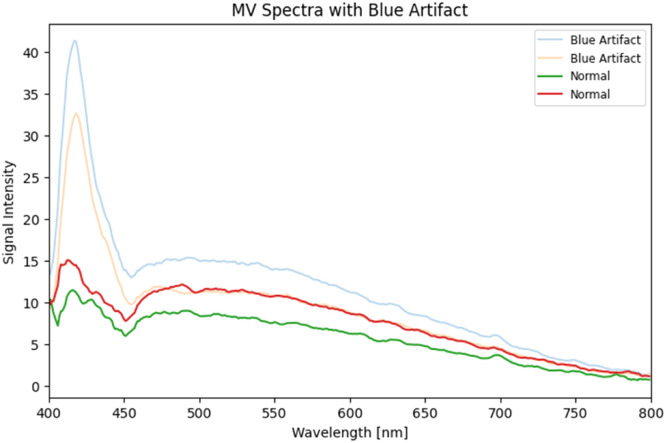
Spectra collected near blue artifacts (faded spectra, ‘Blue Artifact’) and away from the artifact (solid spectra, ‘Normal’).

When taking data near blue artifacts (artifact fills approximately 50% of the closed microscope field diaphragm), the spectra exhibited high signal intensity in the 400–450 *nm* range relative to measurements collected away from artifacts (Fig. 12). That is, the blue artifacts interfered with accurate *λ*_max_ detection, and potentially with RGB calculations, making results unreliable. Therefore, measurements near blue artifacts should be avoided.

### Number of measurements

The analysis of the coefficient of variation (CV) for seven (six for 100 *μm* cable) coal samples—measured under the same conditions—and two shale samples—measured under six different conditions—reveals the optimal number of measurements for achieving reliable and consistent data. Table 2 presents the mean and standard deviation of CV for *λ*_max_ and the R/G quotient under these measurement scenarios.

**Table 2:** Coefficient of variation (CV) and standard deviation of CV for ***λ*_max_** and RG quotients for different numbers of measurements. The top sections A and B present data from six (section A) and seven (section B) coal samples measured under the same conditions using the **100*μm*** (section A) and **200*μm*** (section B) fiber-optic cables. The bottom sections C and D show results from two shale samples measured under six different conditions.

A					B				
Measurements	λ_max_ mean	λ_max_ std	R/G mean	R/G std	Measurements	λ_max_ mean	λ_max_ std	R/G mean	R/G std
5	0.082	0.060	0.159	0.128	5	0.046	0.023	0.086	0.051
10	0.058	0.052	0.146	0.066	10	0.071	0.011	0.104	0.047
15	0.082	0.052	0.292	0.370	15	0.055	0.011	0.100	0.044
20	0.089	0.038	0.136	0.041	20	0.056	0.032	0.110	0.041
25	0.093	0.044	0.238	0.142	25	0.059	0.025	0.133	0.048
C					D				
Measurements	λ_max_ mean	λ_max_ std	R/G mean	R/G std	Measurements	λ_max_ mean	λ_max_ std	R/G mean	R/G std
5	0.004	0.003	0.045	0.021	5	0.045	0.036	0.025	0.013
10	0.010	0.004	0.061	0.028	10	0.087	0.038	0.032	0.011
15	0.012	0.004	0.059	0.022	15	0.057	0.038	0.045	0.030

Based on the results, ten measurements are recommended as the ideal number to balance measurement precision versus the time required for analysis. This is supported by the relatively low standard deviation across multiple conditions, with CV values remaining <0.1, indicating high measurement stability. While fewer than ten measurements may not sufficiently capture sample variability, increasing the number of measurements beyond fifteen provided diminishing returns. Thus, ten measurements offered the best compromise between statistical reliability and measurement efficiency for spectral fluorescence analysis from the data collected in this study. This is consistent with prior protocols noted for measurement of fluorescence emission wavelength of SOM [13], although recent studies have recommended 15 to 20 individual measurements per sample [18].

### Effect of measurement number on CV

A one-way ANOVA was first performed to determine whether the number of measurements had a statistically significant effect on the coefficient of variation (CV) for *λ*_max_and R/G quotient. The results are summarized in Table 3. Most datasets showed no statistically significant difference among the measurement-number groups at the α=0.05 level. This indicates that, for most cases, changing the number of measurements did not produce a statistically significant change in CV. However, one significant ANOVA result was observed for $CV_{\lambda_{\max\;}}$for shale sample 1, *F* = 4.494, *p* = 0.030. This indicates that the number of measurements had a statistically significant effect on the $CV_{\lambda_{\max\;}}$for this shale dataset. No significant effects were observed for the coal datasets or for the R/G quotient datasets.

**Table 3:** One-way ANOVA results for the effect of measurement number on CV for both coal and shale samples, where *F* is test statistic, p-value is compared with the significant level, *α* = 0.05, and the significant column shows if rejecting the null hypothesis is necessary.

Dataset	F	p-value	Significant
Coal 100 μm λ_max_	0.546	0.704	No
Coal 200 μm λ_max_	0.654	0.629	No
Coal 100 μm RG	0.714	0.591	No
Coal 200 μm RG	0.868	0.495	No
Shale Sample 1 λ_max_	4.494	0.030	Yes
Shale Sample 1 RG	0.754	0.487	No
Shale Sample 2 λ_max_	2.557	0.111	No
Shale Sample 2 RG	1.560	0.242	No

#### Pairwise comparison

For the coal samples shown in Table 4, none of the pairwise comparisons were statistically significant after Holm correction. This result was consistent for both the 100 *μm* and 200 *μm* fiber-optic cables for both *λ*_max_ CV and RG quotient CV. In particular, 10 measurements did not differ significantly from 15, 20, or 25 measurements, indicating that increasing data collection beyond 10 measurements did not significantly improve measurement stability.

**Table 4:** Pairwise CV comparison results for coal samples using 10 measurements as reference, where *F* is test statistic, adjusted p-value is compared with the significant level, *α* = 0.05, and the significant column shows if rejecting the null hypothesis is necessary.

Dataset	Comparison	F	p-value	Adjusted p-value	Significant
Coal 100 μm λ_max_	10 vs 5	−0.8783	0.4200	1.0000	No
Coal 100 μm λ_max_	10 vs 15	−0.7137	0.5148	1.0000	No
Coal 100 μm λ_max_	10 vs 20	−1.2436	0.2815	1.0000	No
Coal 100 μm λ_max_	10 vs 25	−1.2174	0.2903	1.0000	No
Coal 100 μm RG	10 vs 5	−0.3253	0.7581	1.0000	No
Coal 100 μm RG	10 vs 15	−1.0223	0.3644	1.0000	No
Coal 100 μm RG	10 vs 20	0.2401	0.8221	1.0000	No
Coal 100 μm RG	10 vs 25	−1.9693	0.1203	0.4811	No
Coal 200 μm λ_max_	10 vs 5	1.2066	0.2730	0.8190	No
Coal 200 μm λ_max_	10 vs 15	1.1094	0.3097	0.8190	No
Coal 200 μm λ_max_	10 vs 20	1.9181	0.1132	0.4528	No
Coal 200 μm λ_max_	10 vs 25	0.8824	0.4179	0.8190	No
Coal 200 μm RG	10 vs 5	1.0085	0.3521	1.0000	No
Coal 200 μm RG	10 vs 15	0.2860	0.7845	1.0000	No
Coal 200 μm RG	10 vs 20	−0.5136	0.6294	1.0000	No
Coal 200 μm RG	10 vs 25	−1.6808	0.1536	0.6146	No

For the shale samples shown in Table 5, none of the pairwise comparisons were statistically significant after Holm correction. Although one comparison, the $CV_{\lambda_{\max\;}}$for shale sample 2 between 10 and 5 measurements, had a raw p-value below 0.05 (*p* = 0.0432), it was not significant after Holm correction $\left(p_{Holm}=0.0864\right)$. Therefore, this comparison was not considered statistically significant after accounting for multiple comparisons.

**Table 5:** Pairwise CV comparison results for shale samples using 10 measurements as reference, where *F* is test statistic, adjusted p-value is compared with the significant level, *α* = 0.05, and the significant column shows if rejecting the null hypothesis is necessary.

Dataset	Comparison	F	p-value	Adjusted p-value	Significant
Shale Sample 1 λ_max_	10 vs 5	2.1781	0.0649	0.1299	No
Shale Sample 1 λ_max_	10 vs 15	−0.4726	0.6481	0.6481	No
Shale Sample 1 RG	10 vs 5	1.0865	0.3049	0.6098	No
Shale Sample 1 RG	10 vs 15	0.1296	0.8996	0.8996	No
Shale Sample 2 λ_max_	10 vs 5	2.3403	0.0432	0.0864	No
Shale Sample 2 λ_max_	10 vs 15	1.6363	0.1361	0.1361	No
Shale Sample 2 RG	10 vs 5	1.0362	0.3254	0.6508	No
Shale Sample 2 RG	10 vs 15	−0.9922	0.3581	0.6508	No

#### Summary of measurement number selection

The ANOVA results showed that only $CV_{\lambda_{\max\;}}$for shale sample 1 had a significant overall difference among measurement-number groups. However, the pairwise comparisons using 10 measurements as the reference showed no statistically significant differences after Holm correction for any dataset. This indicates that the result from 10 measurements was statistically comparable to the other measurement number groups after correcting for multiple comparisons. For the coal samples, the result from 10 measurements was not significantly different from 15, 20, or 25 measurements. This supports the conclusion that increasing data collection beyond 10 measurements did not significantly improve measurement stability. For the shale samples, the result from 10 measurements also was not significantly different from 5 or 15 measurements after Holm correction, including the $CV_{\lambda_{\max\;}}$for shale sample 1 that showed a significant overall ANOVA result.

Therefore, collection of 10 measurements per sample was determined as a sufficient measurement number for the analysis. This determination was based on the observation that 10 measurements produced CV values statistically comparable to larger measurement counts while reducing measurement time. The results do not indicate that 10 measurements always produced the lowest CV, but they suggest that 10 measurements provided a practical balance between measurement stability and experimental efficiency.

### Interpolation

**Fig. 13:**
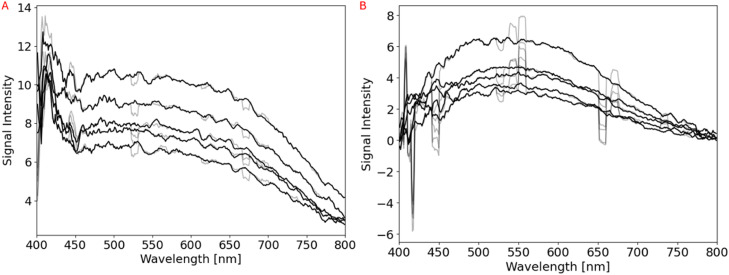
Interpolated spectra are presented in solid color lines, and raw data with a 9 nm moving average window in faded lines, for a shale sample (panel A) and a coal sample (panel B).

The implementation of interpolation smoothed artifacts and resulted in more reproducible *λ*_max_ and RGB quotient values. The Fossil software applies a moving average (MV, using a 9 nm window) to remove artifacts (Fig. 13A-B). The interpolation used in this study replaced the MV approach to remove artifact spikes and dips in raw spectra using the average of the adjacent preceding and following data points.

**Fig. 14:**
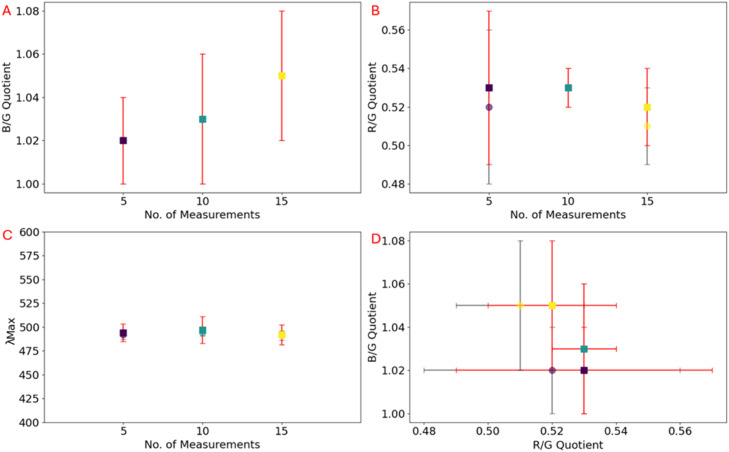
Comparison between raw data (faded grey error bars with circular symbols show standard deviation) and interpolated data (solid red error bars with square symbols show standard deviation) for a shale sample using the **200*μm*** fiber-optic cable. Different color symbols correspond to 5, 10, and 15 measurements. A. B/G quotient. B. R/G quotient. C. ***λ*_max_**. D. B/G quotient versus R/G quotient. Raw data and interpolated data in panel A show precise overlap in ratio and error.

**Fig. 15:**
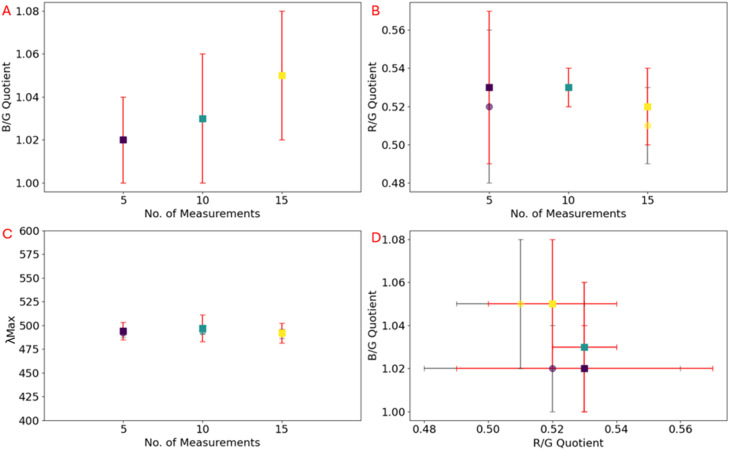
Comparison between raw data (faded grey error with circular symbols bars show standard deviation) and interpolated data (solid red error bars with square symbols show standard deviation) on a coal sample using the **200*μm*** fiber-optic cable. Different color symbols correspond to 5, 10, and 15 measurements. A. B/G quotient. B. R/G quotient. C. ***λ*_max_**. D. B/G quotient versus R/G quotient.

**Fig. 16:**
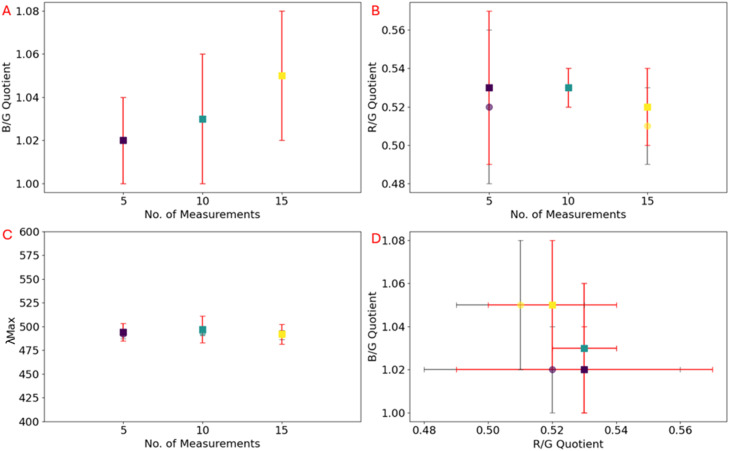
Comparison between raw data (faded grey error bars with circular symbols show standard deviation) and interpolated data (solid red error bars with square symbols show standard deviation) for a coal sample using the **100*μm*** fiber-optic cable. Different color symbols correspond to 5, 10, and 15 measurements. A. B/G quotient. B. R/G quotient. C. ***λ*_max_**. D. B/G quotient versus R/G quotient.

It is evident that interpolation enhanced the smoothness of spectra and improved consistency in detection of *λ*_max_ position and calculation of RGB quotients when impacted by artifacts (Fig. 14). Generally, the variation in RGB quotients between pre- and post-interpolated data is minimal, typically within a range of 0.01–0.02, and *λ*_max_ position also is highly consistent, suggesting that interpolation does not substantially distort fluorescence parameters and can be used as a valid post-processing step when necessary. However, interpolation also can be observed to influence data reduction in situations where artifact spectrometer noise directly affected the calculation of RGB quotient or detection of *λ*_max_ position. For example, interpolated smoothing of artifact troughs at 650–660 nm for the coal sample (Fig. 13B) resulted in systematically greater R/G values (Fig. 15B, D, Fig. 16B, D). Therefore, if necessary to remove artifacts, interpolation should be applied uniformly across all data in a study to maintain comparability and should be explicitly acknowledged in data reporting.

### BAM secondary validation

A secondary validation analysis was conducted utilizing the BAM-F012 glass reference slide to confirm that measured spectral peaks were within ±5 nm of the certified reference values (Figure).

Based on the displayed spectra, the measurements are consistent with the expected reference profile provided by Bundesanstalt für Materialforschung und -prüfung [16]. Table 6 summarizes the eight major spectral peaks, including their measured *λ*_max_ values and normalized intensities, which serve as a benchmark for evaluating the system’s accuracy.

**Table 6:** Summary of major spectral peaks from BAM-F012 spectra [16] (left) and measured average result (***n* = 10**, right), including the peak wavelength (***λ*_max_**), and normalized intensity.

Expected Data		Experimental Data	
Peak (nm)	Normalized Intensity (I)	Peak (nm)	Normalized Intensity(I)
488	0.0288	489	0.0516
542	0.1280	544	0.1620
548	0.1085	544	0.1620
591	0.9090	591	0.0871
612	0.3070	612	0.2538
620	0.2070	617	0.1953
653	0.0185	648	0.0183
701	0.1120	697	0.0699

**Fig. 17:**
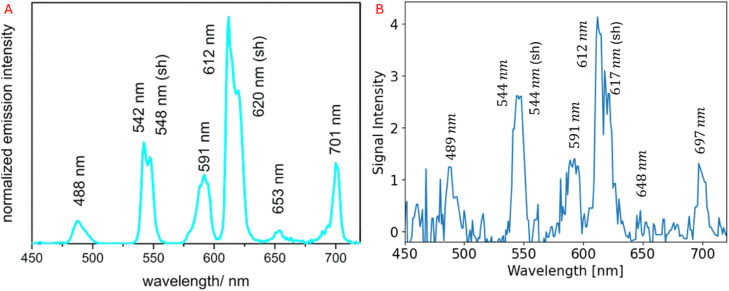
A. Emission spectrum from the certified reference material (BAM-F012), showing eight major spectral bands and shoulders (sh). Excitation for the certified reference spectrum was performed at **365*nm***. B. Averaged spectrum on ten spectra collected from the BAM-F012 reference material using the method outlined herein, including interpolation. The displayed spectra align closely with the reference spectrum [16].

According to Fig. 17 and Table 6, the most prominent peak was detected at 612 *nm*, matching the *λ*_max_ reported by Bundesanstalt für Materialforschung und -prüfung [16]. Furthermore, all major peaks fell within the ±5 *nm* tolerance threshold and normalized intensity values for all peaks were acceptable, indicating the spectrometer is functioning reliably. Also apparent in Fig. 17B is significant noise as compared to the reference spectrum in Fig. 17A. This makes evaluation of smaller peaks (e.g., 653 nm) more difficult, and highlights limitations of the tested system. Nevertheless, this test clearly demonstrated that secondary validation via measurement of the BAM-F012 standard reference material served as a dependable method for independently verifying spectrometer accuracy for fluorescence-based measurements.

## Limitations

This method is limited to liptinite organic matter types (sporinite, telalginite, lamalginite, bituminite) with high to moderate fluorescence emission intensity occurring in geologic samples (coal and shale) which range from immature to mature for oil generation in thermal maturity (overall approximate vitrinite reflectance range 0.3% to 1.1%). This method is not applicable to geologic samples of higher thermal maturity where fluorescence emission intensity is reduced or absent and is restricted exclusively to samples which contain fluorescent organic matter types. Although the calibration lamp utilizes the highly stable PTFE fluoropolymer, there are no data yet available to monitor for the potential of long-term deterioration of the PTFE film, nor are data yet available for its measurement comparison in interlaboratory exercises. Long-term monitoring of measurement stability for the PTFE calibration lamp and the BAM standard, and the interlaboratory comparison of their measurement, could be performed as future checks on these limitations. Apart from introducing recommendations for the number of sample measurements and the conditions for measurement, this method investigation introduces an improvement via the use of widely available PTFE as a calibration standard. The prior calibration method utilized a unique opaline quartz lamp [9] which is no longer commercially available. However, there are no data yet available to ascertain the reductions in measurement error and improvement in CV potentially represented by the use of a PTFE versus opaline quartz lamp. Future studies could also quantify these potential improvements.

## Ethics statements

Not applicable.

## Related research article

None, in preparation.

## CRediT author statement

**Kavin Siaw:** Analysis Data acquisition, Software, Validity tests, Writing – original draft preparation. **Paul Hackley:** Conceptualization, Methodology, Writing.

## Declaration of competing interest

The authors declare that they have no known competing financial interests or personal relationships that could have appeared to influence the work reported in this paper.

## Data Availability

The data are contained in tables in the manuscript and are publicly available online from release.https://doi.org/10.5066/P13KZXXF.a
